# *Fusobacterium nucleatum* drives CD40-mediated dendritic cell activation and Th17/Treg imbalance to exacerbate intestinal inflammation in Crohn’s disease

**DOI:** 10.3389/fimmu.2025.1712971

**Published:** 2026-01-06

**Authors:** Mingyuan Wang, Junjian Sun, Jiang Yu, Jiayun Wang, Chenjing Xu, Jingjing Ma, Hongjie Zhang

**Affiliations:** 1 Department of Gastroenterology, The First Affiliated Hospital with Nanjing Medical University, Nanjing, China; 2 Department of Gastroenterology, Nanjing First Hospital, Nanjing Medical University, Nanjing, China

**Keywords:** CD40, Crohn’s disease, dendritic cells, *Fusobacterium nucleatum*, Th17/Treg balance

## Abstract

**Background:**

Crohn’s disease (CD) is a chronic relapsing inflammatory bowel disease characterized by persistent mucosal inflammation and immune dysregulation. While alterations in gut microbial composition are known to contribute to CD pathogenesis, the precise mechanisms linking specific microbial species to immune dysfunction remain unclear. Here, we identify *Fusobacterium nucleatum* (*Fn*), a pathobiont enriched in CD patients, as a key driver of dendritic cell (DC) activation and downstream Th17/Treg imbalance.

**Methods:**

Fecal and colonic mucosal samples from CD patients and healthy controls were analyzed by 16S rRNA sequencing. A TNBS-induced colitis model with adoptive DC transfer, was used to evaluate the impact of *Fn* on intestinal inflammation, DC activation and Th17/Treg balance. RNA sequencing of *Fn*-exposed bone marrow-derived DCs (BMDCs) identified key immune mediators. CD40 signaling was verified through inhibition with TRAF-STOP, both *in vivo* and *in vitro*.

**Results:**

*Fn* was significantly enriched in CD and positively associated with severity of inflammation. In mice, *Fn* aggravated colitis, resulting in heightened immune dysregulation, especially DC activation. Adoptive transfer of *Fn*-primed DCs aggravated TNBS-induced inflammation in recipient mice, accompanied by Th17/Treg imbalance. Transcriptomic analysis identified robust CD40 upregulation in *Fn*-exposed DCs, which was corroborated in inflamed colons from CD patients. CD40 blockade with TRAF-STOP suppressed DC activation, restored Th17/Treg balance, and ameliorated intestinal inflammation.

**Conclusions:**

This study demonstrates that *Fn* exacerbates intestinal inflammation under inflammatory conditions in CD through CD40-mediated DC activation and subsequent Th17/Treg imbalance. These findings establish a novel mechanistic link between microbial dysbiosis and immune dysregulation, identifying CD40 as a promising target for microbiota-directed immunotherapy.

## Introduction

Crohn’s disease (CD) is a chronic inflammatory disorder of the gastrointestinal tract characterized by gut inflammation and immune imbalance, driven by aberrant microbiota–host interactions (1). Despite advances in understanding its pathogenesis, a significant proportion of patients experience persistent mucosal damage and disease progression, underscoring the need for targeted therapeutic strategies (2). A hallmark of CD is dysbiosis, with advances in microbiome research systematically revealing the compositional and functional aberrations of intestinal microbiota in CD (3, 4). Among the microbial taxa altered, *Fn* has emerged as a notable pathobiont, previously implicated in colorectal carcinogenesis (5, 6). However, its enrichment and immunomodulatory contributions to intestinal inflammation in CD, remain poorly defined.

Professional antigen-presenting cells (APCs) serve as immunological gatekeepers at the mucosal interface, with dendritic cells being the most powerful APCs in initiating immune responses (7). Through pattern recognition receptor (PRR)-mediated microbial sensing, DCs coordinate innate-adaptive immunity crosstalk by modulating T cell polarization and cytokine networks (8). Under homeostasis, DCs maintain immune tolerance by promoting regulatory T cell (Treg) differentiation and suppressing T helper 17 (Th17) responses (9, 10). However, upon activation by pathogenic stimuli or microbial dysbiosis, DCs undergo a functional shift toward a pro-inflammatory phenotype mediated by upregulating co-stimulatory molecules (e.g., CD80, CD86) and secreting cytokines (IL-6, TNF-α), amplifying Th17-driven inflammatory cascades (11–13). Th17 cells, characterized by their secretion of pro-inflammatory cytokines (IL-17, IL-22), contribute to mucosal barrier disruption and perpetuation of intestinal inflammation in CD. In contrast, Treg serve as critical anti-inflammatory regulators that secrete immunosuppressive cytokines (IL-10, TGF-β) to sustain immune tolerance. The Th17/Treg balance, tightly regulated by DC-mediated signals, is pivotal for determining intestinal inflammatory outcomes (14–16). In CD, dysfunctional DC activation disrupts the Th17/Treg balance, promoting enhanced Th17 responses while impairing Treg differentiation, thereby exacerbating intestinal inflammation. However, the upstream microbial cues and receptor-mediated pathways driving aberrant DC activation and consequent Th17/Treg imbalance in CD remain poorly defined.

CD40, a key costimulatory receptor on DCs, integrates microbial and immune signals to regulate DC activation and T cell priming (17, 18). In CD, elevated CD40 expression is associated with mucosal immune dysregulation and disease severity (19, 20). Although CD40 engagement activates multiple downstream pathways, including NF-κB, its role in driving adaptive immune imbalances, particularly Th17/Treg skewing, remains to be fully elucidated. Whether *Fn*, an enriched pathobiont in CD-associated dysbiosis, aggravates intestinal inflammation through CD40-dependent DC activation and subsequent Th17/Treg imbalance remains unclear.

Here, we integrate clinical analyses, murine colitis models, and mechanistic studies to investigate how *Fn* promotes CD40-dependent DC activation and drives Th17/Treg imbalance in CD. We show that *Fn* upregulates CD40 on DCs, driving their maturation and exacerbating intestinal inflammation. Moreover, *Fn*-induced DCs disrupt Th17/Treg balance, contributing to mucosal immune dysregulation. Pharmacological inhibition of CD40 restores DC homeostasis and normalizes Th17/Treg balance. These findings uncover a novel microbial-immune axis linking dysbiosis to adaptive immune imbalance in CD and highlight CD40 as a potential therapeutic target.

## Materials and methods

### Human samples collection

A total of 24 CD patients and 15 healthy subjects (HSs) were recruited at the gastroenterology department of the First Affiliated Hospital with Nanjing Medical University from April 2021 to April 2022. Fecal and colonic mucosal samples were collected from each participant. This study was approved by the Ethics Committee of the First Affiliated Hospital with Nanjing Medical University (Approval No. 2021-SR-312). Written informed consent was obtained from all participants prior to enrollment. Demographic and clinical characteristics, including age, gender, disease location/behavior (Montreal classification) and CDAI, were recorded and are summarized in **Supplementary Table S1**.

### 16S rRNA gene amplicon sequencing

Total microbial DNA was extracted using the CTAB method, eluted in 50 μL of elution buffer and stored at -80°C. The V3-V4 regions of 16S rRNA genes were amplified using primers 341F: (5’-CCTACGGGNGGCWGCAG-3’) and 805R: (5’-GACTACHVGGGTATCTAATCC-3’), with sample-specific barcodes. PCR reactions (25 μL) contained 25 ng DNA, 12.5 μL PCR Premix, primers, and PCR-grade water. Cycling conditions were: 98°C for 30 seconds; 32 cycles of 98°C for 10 seconds, 54°C for 30 seconds, 72°C for 45 seconds; and final extension at 72°C for 10 minutes. PCR products were confirmed with 2% agarose gel electrophoresis, purified by AMPure XT beads (Beckman Coulter Genomics, Danvers, MA, USA) and quantified by Qubit (Invitrogen, Carlsbad, CA, USA). The libraries were sequenced on the NovaSeq PE250 platform.

Raw paired-end reads were assigned to samples by barcode, merged with FLASH, and filtered using fqtrim (v0.94). Chimeric sequences were filtered using Vsearch (v2.3.4), and features were dereplicated with DADA2 to generate feature tables. Taxonomic classification was performed using the SILVA 138 database. Alpha diversity and beta diversity were calculated with QIIME2 and visualized using the R package (v3.5.2). Sequence alignment and annotation were performed using BLAST and SILVA.

### GEO data acquisition

Public gene expression profiles were obtained from the Gene Expression Omnibus (GEO) database. The datasets GSE75214 (21), GSE261086 (22) and GSE52746 (23), which contain transcriptomic data from colonic tissues of healthy controls and CD patients, were retrieved for analysis. Specific information of the study participants has been shown in **Supplementary Table S2**. Differentially expressed genes (DEGs) between groups were identified using the GEO2R web tool, with significance thresholds set according to default statistical criteria (|log2 fold change| > 1.5, *P* < 0.05).

### Mice

Animals used for experiments were C57BL/6 mice, aged 8 weeks and weighing 18-20 g, obtained from the Model Animal Research Center of Nanjing University (Jiangsu, China). All mice were maintained under specific pathogen-free (SPF) conditions in Nanjing Medical University Animal Care Facilities. All animal experiments were approved by the Experimental Animal Care and Ethics Committee of Nanjing Medical University (IACUC-2207038).

### Bone marrow-derived dendritic cells culture and treatments

BMDCs were generated as previously described (24). Bone marrow-derived (BM) cells were fushed from mouse femurs and tibias with RPMI-1640 medium (Gibco, Grand Island, NY, USA), and red blood cells were lysed. Cells were cultured in RPMI-1640 containing 1% penicillin–streptomycin (NCM Biotech, Jiangsu, China) supplemented with 10% fetal bovine serum (FBS, Gibco), 20 ng/mL granulocyte–macrophage colony-stimulating factor (GM-CSF, PeproTech, Rocky Hill, NJ, USA), and 10 ng/mL interleukin-4 (IL-4, PeproTech). On Days 3 and 5, half of the medium was replaced with fresh cytokines. On Day 7, the entire medium was refreshed. The purity of BMDCs was validated by flow cytometry, exceeded 95%, and shown in **Supplementary Figures S2A, B**.

### Bacterial strains and infection

*F. nucleatum* (ATCC 25586) was cultured anaerobically at 37°C. Bacteria in the logarithmic growth phase were harvested, centrifuged at 3,000 × g for 15 minutes, and resuspended in antibiotic-free RPMI-1640. Heat-inactivated *Fn* was prepared at 90°C for 30 minutes (25). BMDCs were co-cultured with *Fn* or heat-inactivated *Fn* (at a multiplicity of infection, MOI = 100:1) under a humidified 5% CO_2_ atmosphere at 37°C for 24 hours. BMDCs co-cultured with *Fn* were referred to as *Fn*-DCs, with the bacterial viability specified in each experimental context.

### Induction of experimental colitis by TNBS

Experimental colitis was induced using 2.5 mg of 2,4,6-trinitrobenzenesulfonic acid (TNBS, Sigma Aldrich, St. Louis, MO, USA) in 50% ethanol, administered 4 cm intrarectally under sodium pentobarbital anesthesia (2%, 45 mg/kg, intraperitoneal injection), as previously described (26). Mice were maintained in a head-down position for 1 minute to prevent drug solution from flowing out. In the groups receiving *Fn*, mice were administered a broad-spectrum antibiotic cocktail (containing ampicillin 0.2 g/L, metronidazole 0.2 g/L, neomycin 0.2 g/L, and vancomycin 0.1 g/L, Sigma Aldrich) in their drinking water for 4 days, followed by daily gavage of *Fn* (1 × 10^8^ CFU per dose) or PBS for 10 days before TNBS-induced colitis. In the CD40 inhibition experiment, *Fn*-treated mice received daily intraperitoneal injections of the CD40-specific inhibitor TRAF-STOP (27) (6877002, MCE, Monmouth Junction, NJ, USA) or an equal volume of vehicle control starting from day 1 of TNBS-induced colitis.

The disease activity index (DAI) was used to evaluate colonic damage including weight loss, stool consistency, and bloody stool, as described previously (28). Histological scores of colonic hematoxylin and eosin (H&E) staining were calculated as described (29).

### Adoptive transfer murine model

1 × 10^6^*Fn*-DCs (BMDCs co-cultured with heat-inactivated *Fn*), control DCs or PBS were adoptively transferred to recipient mice. On Days 0, 2 and 4, PBS, BMDCs, or *Fn*-DCs in a total volume of 100 µL were injected via the tail vein into mice.

### Isolation and flow cytometry detection of colonic lamina propria mononuclear cells

LPMCs were isolated as described (30). Mouse colons were cut into 2 mm segments, incubated in PBS containing 2% FBS, 0.5 mM EDTA (Corning, NY, USA), and 10 mM HEPES (Corning) at 37 °C and 220 rpm for 40 minutes to remove epithelial cells. Tissues were filtered through a 70 μm cell strainer and subsequently digested in pre-warmed RPMI-1640 with 2% FBS, 1% penicillin-streptomycin, 100 U/mL Collagenase IV (Biosharp, Anhui, China) and 2 µg/mL DNase (Sigma Aldrich) at 37°C and 220 rpm for 30 minutes. The suspension was filtered, centrifuged, and subjected to Percoll gradient centrifugation (40%/75%, prepared in PBS, Cytiva, Marlborough, MA, USA) to collect LPMCs. Then LPMCs were incubated with fluorochrome-conjugated antibodies, including PE-CD11c, APC-CD40, PerCP/Cyanine5.5-CD45, FITC-CD80, and PE-Cy™7-CD86 (BioLegend, San Diego, CA, USA) for 30 minutes at 4°C in the dark. The staining procedures for Th17 and Treg cell differentiation were described as follows for MLN cells. Fluorescence minus one (FMO) controls were included as negative controls to aid in gating. Data acquisition was conducted using a FACSCanto II flow cytometer (BD, San Jose, CA, USA), and data were analyzed with FlowJo software version 10.8.1 (TreeStar, Ashland, OR, USA). The detailed gating strategies for analysis are shown in **Supplementary Figures S1A, B**.

### Isolation and flow cytometry detection of mesenteric lymph node cells

MLN cells were isolated by pressing mesenteric lymph nodes through a 70 µm cell strainer (31), resuspended in RPMI-1640 with 10% FBS, and stimulated with cell stimulation cocktail (eBioscience) at 37 °C, 5% CO_2_ for 5 hours to detect Th17 cells. Cells were incubated with PerCP/Cyanine5.5-CD3e and FITC-CD4 (eBioscience) at 4 °C for 30 minutes in the dark, then resuspended in fixation/permeabilization solution (Multi Sciences, Zhejiang, China) and incubated with eFluor™ 450-IL-17A (eBioscience) for 30 minutes at 4 °C in the dark.

For Treg detection, cells were stained with PerCP/Cyanine5.5-CD3e, FITC-CD4 and Alexa Fluor™-CD25 (eBioscience) for 30 minutes at 4 °C in the dark, followed by Foxp3/Transcription Factor Staining Buffer Set (eBioscience), and intracellular staining with PE-Foxp3 (eBioscience). FMO controls were included as negative controls to aid in gating. Flow cytometry was performed using a FACSCanto II flow cytometer (BD), and data were analyzed with FlowJo software version 10.8.1 (TreeStar). The detailed gating strategy for MLN cell analysis is shown in **Supplementary Figure S1B**.

### RNA sequencing

Total RNA was extracted by Trizol (Invitrogen), and mRNA was purified using poly-T oligo-attached magnetic beads. DNA fragments were selectively enriched using Illumina PCR Primer Cocktail. The sequencing library was sequenced on NovaSeq 6000 platform at Shanghai Personal Biotechnology Cp. Ltd. Paired-end clean reads were aligned to the mouse genome version GRCm39 using HISAT2 (v2.1.0). Differential expression was analyzed using DESeq2. *P* < 0.05 and | log2 (fold change) | > 1 were considered significant.

### Western blotting

Colonic tissues or BMDCs were lysed in RIPA lysis buffer (Beyotime, Shanghai, China) supplemented with a protease inhibitor cocktail (MCE). Protein concentrations were determined using a BCA assay (Beyotime). Equal amounts of protein (30 μg per lane) were separated on 10% SDS-PAGE, transferred to PVDF membranes (Millipore, Billerica, MA, USA), and blocked with 5% nonfat milk. Membranes were incubated overnight with antibodies including CD40 specific for human (Cat No. 28158-1-AP, 1:3000, Proteintech, Hubei, China), CD40 specific for mouse (Cat No. 83972-5-RR, 1:5000, Proteintech), p-NF-κB (Cat No. 3033T, 1:1000, CST, Danvers, MA, USA), NF-κB (Cat No. 8242T, 1:1000, CST), p-IκB (Cat No. 9246T, 1:1000, CST), IκB (Cat No. 4812T, 1:1000, CST) and β-actin (Cat No. 66009-1-Ig, 1:10000, Proteintech) at 4 ˚C. Following incubation with HRP-conjugated secondary antibodies (Anti-rabbit IgG-HRP, Cat No. SA00001-2, 1:10000, Proteintech; Anti-mouse IgG-HRP, Cat No. 31430, 1:20000, Thermo Fisher Scientific Inc.), protein bands were visualized using an Enhanced Chemiluminescence reagent (Life Technologies, Gaithersburg, MD, USA), and analyzed using ImageJ software.

### Immunofluorescence staining

Paraffin-embedded sections of human intestinal biopsy specimens were dewaxed and rehydrated using standard protocols, followed by rinsing with tap water. Antigen retrieval was performed by heating the sections in 10 mM citrate buffer for 20 minutes. After blocking for one hour at room temperature, the sections were incubated overnight at 4 °C with antibodies against CD40 (Cat No. 28158-1-AP, 1:200, Proteintech) and CD11c (Cat No. MB65814, 1:100, Bioworld Technology, Bloomington, MN, USA). Subsequently, the sections were incubated with Alexa Fluor 488-conjugated anti-rabbit IgG (Cat No. 111-545-003, 1:1000, Jackson ImmunoResearch, West Grove, PA, USA) and Cy3-conjugated anti-mouse IgG secondary antibodies (Cat No. 115-165-003, 1:1000, Jackson ImmunoResearch) at room temperature for 1 hour. Nuclear staining was then performed with DAPI (Cat No. C1005, Beyotime) for 15 minutes. Finally, the sections were observed and imaged using a Leica THUNDER Imager (Leica Microsystems, Wetzlar, Germany) under consistent exposure settings.

### Quantitative real-time polymerase chain reaction

Total RNA was isolated using TRIzol reagent (Invitrogen) and was reverse transcribed into cDNA. Microbial DNA was extracted using TIANamp Stool DNA Kit (Tiangen, Beijing, China). qRT-PCR was carried out on 7300 Sequence Detection System using ChamQ Universal SYBR qPCR Master Mix (Vazyme Biotech, Nanjing, China). Data were normalized to GAPDH or the universal eubacterial 16S rRNA gene. The primers for the related genes were designed and synthesized by Qingke Biotechnology (Beijing, China). All primers used in this study are listed in **Supplementary Table S3**.

### Statistical analysis

Statistical analysis was performed with SPSS version 22.0 (IBM SPSS Company, Armonk, NY, USA) and GraphPad Prism version 9.0 (GraphPad Software, La Jolla, CA, USA). All experiments were performed at least three times independently, and data are presented as mean ± standard deviation (SD) of biological replicates, unless otherwise stated. For comparisons between two groups, student’s *t*-test was applied while one-way analysis of variance (ANOVA) was adopted for multiple comparisons. *P* < 0.05 was considered statistically significant.

## Results

### *Fn* accumulates in the gut of CD patients

**Figure 1:**
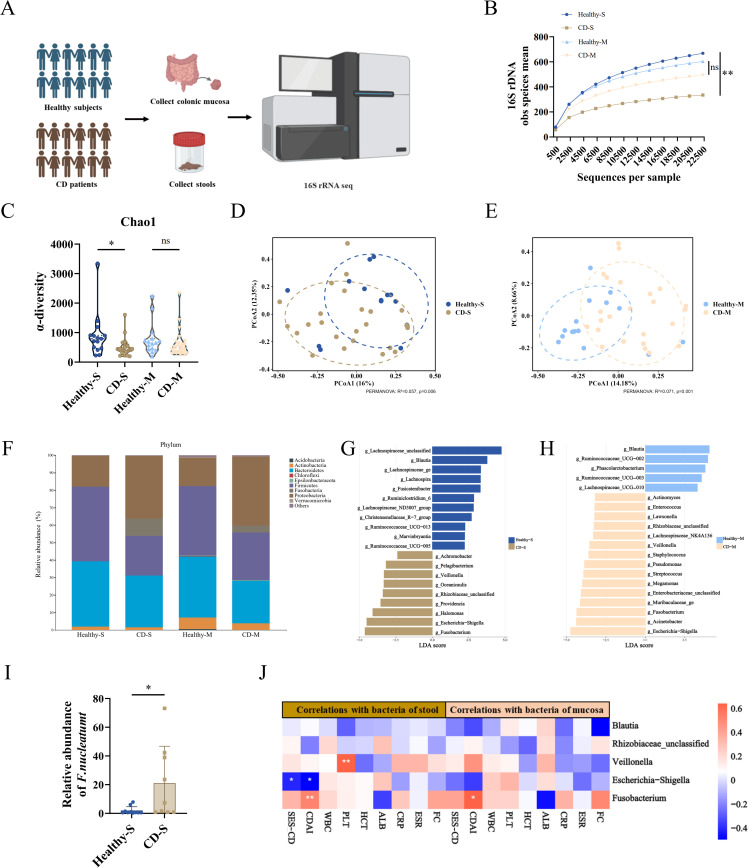
*Fn* accumulates in the gut of CD patients. **(A)** 16S rDNA high-throughput sequencing was performed on bacterial DNA extracted from fecal and colonic mucosal samples of Crohn’s disease (CD) patients (n=24) and healthy subjects (n=15). **(B)** Bacterial diversity was quantified by the mean number of observed species (obs) on the y-axis. **(C)** α-diversity analysis of the aforementioned samples. **(D, E)** β-diversity of microbial communities in fecal and mucosal samples was visualized using unweighted UniFrac-based principal coordinate analysis. **(F)** Phylum-level taxonomic composition analysis of fecal and mucosal microbiota. **(G, H)** Linear discriminant analysis (LDA) identified the most differentially abundant microbial taxa between fecal and mucosal samples. **(I)** Relative abundance of *F.nucleatum* in fecal samples from CD patients versus healthy subjects. **(J)** Spearman correlation analysis between relative bacterial abundance (fecal/mucosal) and clinical parameters (e.g., CDAI, SES-CD) in CD patients. *P < 0.05, **P < 0.01, ***P < 0.001; ns, not significant.

To characterize microbial dysbiosis patterns in CD patients, the intestinal microbiota composition of CD patients (n=24) and healthy subjects (n=15) were analyzed using 16S rRNA gene sequencing. Clinical and demographic characteristics of the enrolled patients are summarized in **Supplementary Table S1**. Baseline variables, including gender, age, BMI, and smoking status, showed no statistically significant differences between CD patients and healthy controls, minimizing the likelihood of demographic bias. Both colonic mucosal and fecal samples were collected from each participant for analysis (**Figure 1A**). Rarefaction curves revealed significantly diminished bacterial diversity in CD fecal microbiota compared to healthy subjects (**Figure 1B**), while a similar trend was observed in mucosal samples, though it did not reach statistical significance (**Figure 1B**). This observation was corroborated by α-diversity (Chao1 index), which consistently revealed reduced microbial richness in CD patients (**Figure 1C**). Principal coordinate analysis (PCoA) of the microbiome revealed significant clustering and separation of microbial communities in both fecal and mucosal samples from CD patients compared to healthy subjects (**Figures 1D, E**).

Taxonomic profiling at the phylum level showed CD fecal microbiota exhibited decreased proportions of Firmicutes and Bacteroidetes, concomitant with enrichment of Proteobacteria and Fusobacteria (**Figure 1F**). Mucosal samples mirrored this microbial dysbiosis pattern, with Proteobacteria being dominant followed by Firmicutes and Bacteroidetes. Notably, a linear discriminant analysis of effect size (LEfSe) identified *Fusobacterium* as the most differentially enriched genus in CD across both sample types (**Figures 1G, H**). Fecal qPCR analysis showed a significantly higher *Fn* abundance in CD patients (**Figure 1I**). To explore the clinical relevance of *Fn* enrichment, we next performed correlation analysis, which revealed strong positive associations between *Fn* levels and disease activity indices, particularly the Crohn’s Disease Activity Index (CDAI) (**Figure 1J**). Collectively, these microbiome profiling data establish *Fn* as a hallmark microbiota signature in CD, with its intestinal accumulation tightly linked to clinical disease activity.

### *Fn* exacerbates TNBS-induced colitis in mice

**Figure 2:**
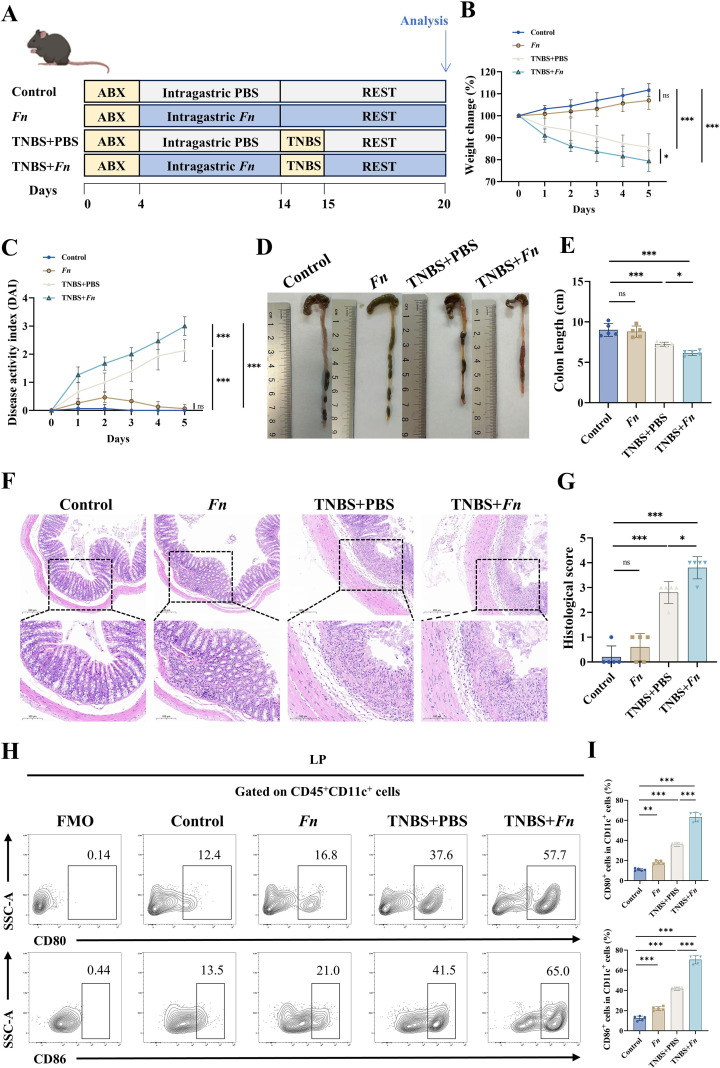
*Fn* exacerbates TNBS-induced colitis in mice. C57BL/6 mice were induced to acute colitis by TNBS after being treated with PBS or infected with *Fn* (Control, n = 5 mice, *Fn*, n = 5 mice, TNBS+PBS, n = 5 mice, and TNBS+*Fn*, n = 5 mice). **(A)** The illustration demonstrates the experiment procedures. **(B)** Mice were weighed daily. **(C)** Disease activity index (DAI) for experimental colitis mice. The general appearance **(D)** and lengths of colons **(E)** in each group. **(F)** Representative images of H&E staining of colon sections, scale bar = 200 μm (above row) and scale bar = 100 µm (below row), and histological scores **(G)** were analyzed. **(H)** Representative flow cytometry plots showing CD80^+^ and CD86^+^ expression on CD11c^+^ DCs within colonic LPMCs. **(I)** Quantification of CD80^+^ and CD86^+^ cells among CD11c^+^ DCs in the colon, shown as bar graphs. *P < 0.05, **P < 0.01, ***P < 0.001; ns, not significant.

To evaluate the pro-inflammatory effects of *Fn*, we established a TNBS-induced murine colitis model comprising four experimental groups: a healthy control group, a group receiving *Fn* alone, a TNBS-only group, and a co-treatment group receiving both TNBS and *Fn* (**Figure 2A**). No significant difference in body weight was observed between mice receiving *Fn* alone and control mice. However, in the context of TNBS-induced colitis, *Fn* resulted in a significant reduction in body weight, indicating a pronounced pro-inflammatory effect (**Figure 2B**). Similarly, while receiving *Fn* alone did not alter disease progression (assessed by DAI), combined treatment with *Fn* and TNBS exacerbated colitis severity, as evidenced by a marked increase in DAI scores (**Figure 2C**).

Mice in the TNBS-only group exhibited marked colonic shortening, which was further exacerbated in the TNBS+*Fn* group compared to TNBS treatment alone (**Figures 2D, E**). Furthermore, H&E staining revealed that combined *Fn* and TNBS treatment induced more severe architectural disruption, exceeding the injury observed in the TNBS group (**Figure 2F**). These findings were further supported by significantly elevated histological scores (**Figure 2G**). Collectively, these findings demonstrate that *Fn* potentiates TNBS-driven gut inflammation, exacerbating both macroscopic and microscopic features of colitis.

### *Fn* exacerbates TNBS-induced colitis by promoting DC activation and Th17/Treg imbalance

As dendritic cells bridge innate microbial sensing and adaptive immune responses, we investigated how *Fn* modulates DC activation and downstream T cell differentiation in TNBS-induced colitis. *Fn* alone modestly increased the proportion of CD80^+^CD86^+^ DCs in the colonic lamina propria compared to controls (**Figures 2H, I**), suggesting a baseline activating effect of *Fn* on DCs. TNBS alone significantly elevated CD80^+^CD86^+^ DC levels, consistent with inflammation-induced DC activation. Notably, in TNBS-treated mice, *Fn* gavage further augmented the proportion of activated DCs beyond levels observed in the TNBS+PBS group, indicating that *Fn* exacerbates DC activation under inflammatory conditions (**Figures 2H, I**).

**Figure 3:**
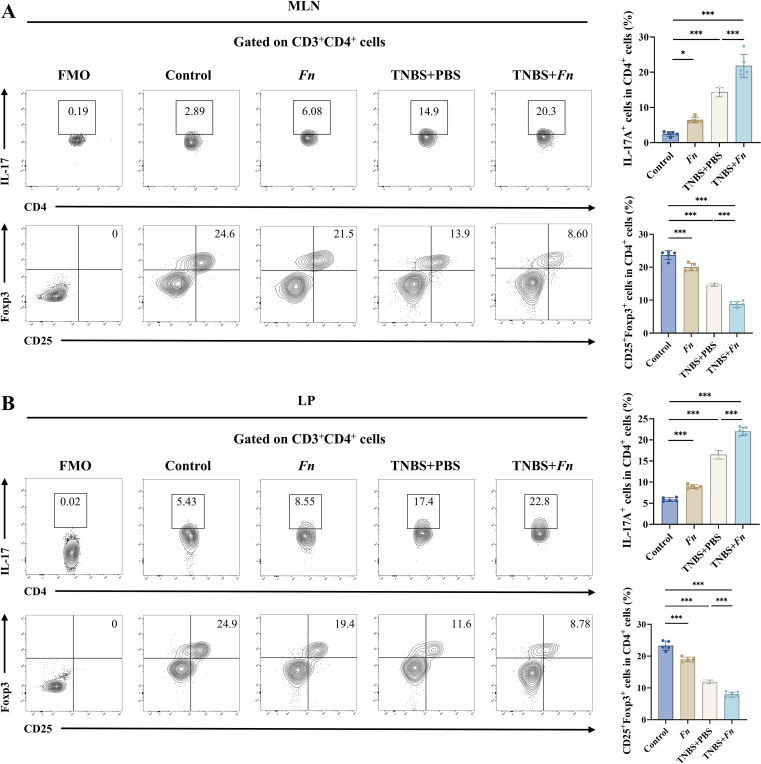
*Fn* exacerbates gut inflammation via dysregulated Th17/Treg differentiation. **(A)** Representative flow cytometry plots showing IL-17A^+^ or CD25^+^Foxp3^+^ expression on CD4^+^ T cells within MLN cells. Quantification of IL-17A^+^ or CD25^+^Foxp3^+^ cells among CD4^+^ T cells in the MLNs, shown as bar graphs. **(B)** Representative flow cytometry plots showing IL-17A^+^ or CD25^+^Foxp3^+^ expression on CD4^+^ T cells within LP cells. Quantification of IL-17A^+^ or CD25^+^Foxp3^+^ cells among CD4^+^ T cells in the colonic LP, shown as bar graphs. *P < 0.05, **P < 0.01, ***P < 0.001; ns, not significant.

Given that activated DCs are key modulators of T cell polarization, particularly in shaping the Th17/Treg balance, we next assessed Th17/Treg differentiation in mesenteric lymph nodes and colonic lamina propria. *Fn* alone induced a limited shift toward increased CD4^+^IL-17A^+^ Th17 cells and decreased CD4^+^CD25^+^Foxp3^+^ Treg cells relative to controls, while TNBS treatment markedly increased Th17 proportions and reduced Treg frequencies. Importantly, *Fn* in TNBS-treated mice further exacerbated this Th17/Treg skewing, with significantly higher Th17 frequencies and lower Treg levels compared to TNBS+PBS controls (**Figures 3A, B**). These findings suggest that the aggravated colitis observed in *Fn*-treated mice is associated with an *Fn*-driven amplification of Th17/Treg imbalance.

To further verify the *in vivo* findings and assess whether *Fn* directly enhances DC activation, BMDCs were stimulated with *Fn*. Consistent with the results observed in intestinal DCs, *Fn* exposure markedly increased CD80/CD86 expression on BMDCs (**Supplementary Figures S2C, D**), confirming its direct pro-maturation effect on DCs in the absence of intestinal environmental cues.

### Adoptive transfer of *Fn*-primed dendritic cells exacerbates TNBS-induced colitis through Th17/Treg imbalance

**Figure 4:**
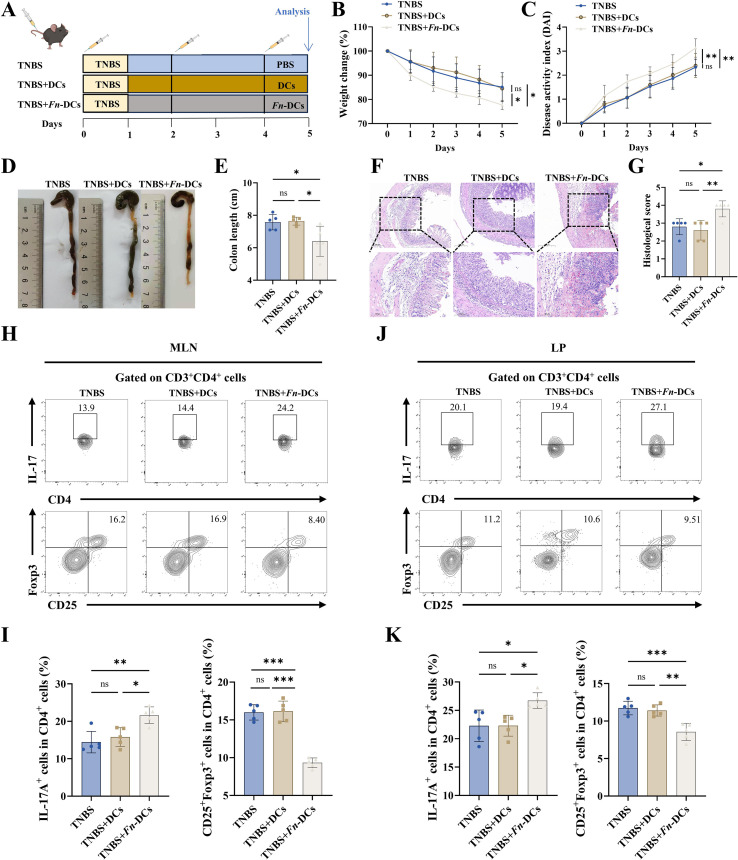
Adoptive transfer of *Fn*-treated dendritic cells promotes Th17/Treg imbalance and aggravates colitis. Colitis was chemically induced in C57BL/6 mice using TNBS. On days 0, 2 and 4, mice received intravenous injections via the tail vein of either PBS, untreated BMDCs, or *Fn-*DCs (BMDCs co-cultured with heat-inactivated *Fn*) (TNBS, n=5 mice, TNBS+DCs, n=5 mice, TNBS+*Fn*-DCs, n=5 mice). **(A)** The schematic demonstrates the experiment procedures. **(B)** Mice were weighed once per day. **(C)** Disease activity index (DAI) for experimental colitis mice. The general appearance **(D)** and lengths of colons **(E)** in each group. **(F)** Representative images of H&E staining of colon sections, scale bar = 200 µm (above row) and scale bar = 100 µm (below row), and histological scores **(G)** were analyzed. **(H)** Representative flow cytometry plots showing IL-17A^+^ or CD25^+^Foxp3^+^ expression on CD4^+^ T cells within MLN cells. **(I)** Quantification of IL-17A^+^ or CD25^+^Foxp3^+^ cells among CD4^+^ T cells in the MLNs, shown as bar graphs. **(J)** Representative flow cytometry plots showing IL-17A^+^ or CD25^+^Foxp3^+^ expression on CD4^+^ T cells within LP cells. **(K)** Quantification of IL-17A^+^ or CD25^+^Foxp3^+^ cells among CD4^+^ T cells in the colonic LP, shown as bar graphs. *P < 0.05, **P < 0.01, ***P < 0.001; ns: not significant.

To confirm the functions of *Fn*-DCs *in vivo*, we adoptively transferred *Fn*-DCs into TNBS-induced colitis mice (**Figure 4A**). Strikingly, recipients of *Fn*-DCs exhibited aggravated colitis compared to mice receiving untreated DCs or PBS, with accelerated body weight loss (**Figure 4B**), colon shortening (**Figures 4D, E**), elevated DAI scores (**Figure 4C**), and more severe histological damage, including transmural ulceration, crypt abscess formation, and inflammatory infiltration (**Figures 4F, G**). These findings demonstrate that *Fn*-primed DCs are sufficient to exacerbate TNBS-induced intestinal inflammation, underscoring their central role as mediators of *Fn*-driven mucosal immune dysregulation. To further explore how *Fn*-primed DCs modulate adaptive immunity, we assessed the Th17/Treg balance. As shown in **Figures 4H-K**, compared to mice injected with PBS or untreated DCs, *Fn*-DC recipients exhibited a significant increase in Th17 cells and a marked decrease in Treg cells. These results indicate that *Fn* promotes excessive DC activation, which in turn drives Th17/Treg imbalance and exacerbates intestinal inflammation under TNBS-induced inflammatory conditions.

### *Fn* induces CD40 upregulation on dendritic cells in murine and human colitis

**Figure 5:**
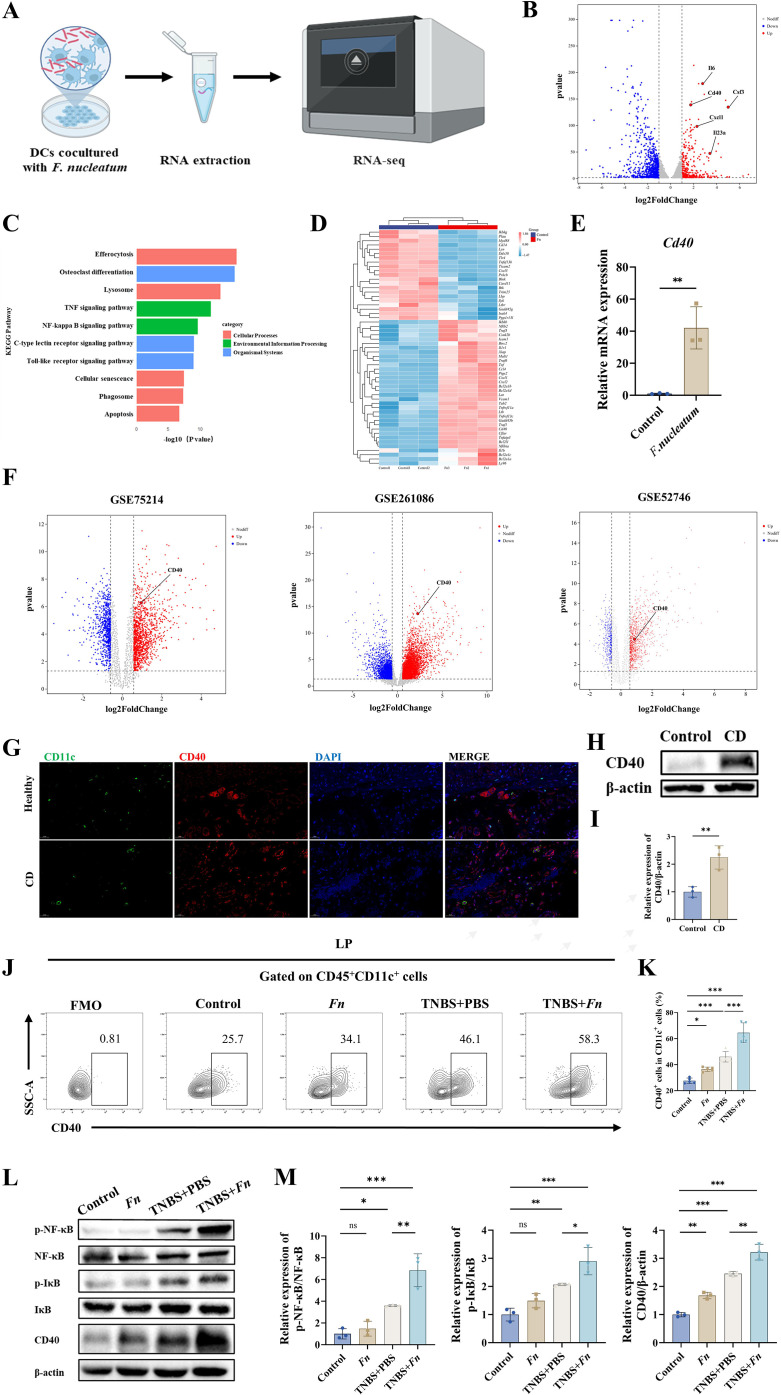
*Fn* exacerbates colitis via CD40-mediated dendritic cells in CD. **(A)** Transcriptomic profiling of BMDCs treated with *Fn* versus untreated controls was performed. **(B)** The volcano plot visualizes the distribution of DEGs. **(C)** The KEGG analysis. **(D)** A heatmap of DEGs enriched in the NF-κB signaling pathway. **(E)** Cd40 was detected by qRT-PCR. **(F)** Volcano plots of DEGs between CD patients and healthy subjects in datasets GSE75214, GSE261086, and GSE52746 in the GEO database. **(G)** Immunofluorescence staining of CD11c and CD40 in human colonic mucosal tissues. **(H)** Representative Western blot showing CD40 protein expression in colonic tissues, with β-actin used as a loading control. **(I)** Quantification of CD40 protein levels from **(H)**, presented as a bar graph. **(J)** Flow cytometry was performed to quantify the proportion of CD40^+^ DCs within colonic LPMCs. **(K)** Statistical significance between groups was determined. **(L)** Representative Western blot showing the expression levels of CD40, phosphorylated NF-κB (p-NF-κB), total NF-κB, phosphorylated IκB (p-IκB), total IκB, and β-actin in colonic tissues. **(M)** Quantification of protein expression levels shown in **(M)**, with statistical comparisons between groups. *P < 0.05, **P < 0.01, ***P < 0.001; ns: not significant.

To explore the molecular mechanism underlying *Fn*-induced dysregulated DC activation, we conducted transcriptomic profiling of BMDCs co-cultured with or without *Fn* (**Figure 5A**). RNA-seq revealed 1,168 DEGs (|fold change| > 2, *P* < 0.05), with pro-inflammatory mediators prominently upregulated (**Figure 5B**). KEGG pathway analysis identified several inflammatory pathways, including NF-κB (**Figure 5C**). Notably, CD40 emerged as one of the top-ranked DEGs (fold change >3, *P* = 1.66×10^−138^), strongly implicating its pivotal role in *Fn*-driven DC activation (**Figure 5D**). qPCR confirmed Cd40 upregulation in *Fn*-stimulated BMDCs (**Figure 5E**).

To evaluate whether these findings are recapitulated in human diseases, we analyzed three independent transcriptomic datasets of CD (GSE75214, GSE261086, GSE52746), all of which consistently demonstrated significant CD40 overexpression in colonic mucosa from CD patients compared to healthy controls (**Figure 5F**). At the protein level, Western blot confirmed elevated CD40 expression in colonic tissues from CD patients (**Figures 5H, I**). Furthermore, immunofluorescence staining of intestinal biopsies revealed that CD40 overexpression was specifically localized to mucosal dendritic cells in CD patients (**Figure 5G**). These results highlight CD40 as a key mediator linking *Fn* exposure to DC-driven immune dysregulation in CD.

To further validate these findings *in vivo*, we isolated LP cells and observed a significant increase in CD40^+^ DCs in *Fn*-treated mice, both with and without TNBS-induced colitis (**Figures 5J, K**). Correspondingly, CD40 protein levels were markedly elevated in TNBS+*Fn* mice, accompanied by enhanced phosphorylation of downstream signaling molecules, including components of the NF-κB pathway (**Figures 5L, M**).

### CD40 inhibition alleviates *Fn*-exacerbated colitis by restoring dendritic cell and Th17/Treg immune balance

**Figure 6:**
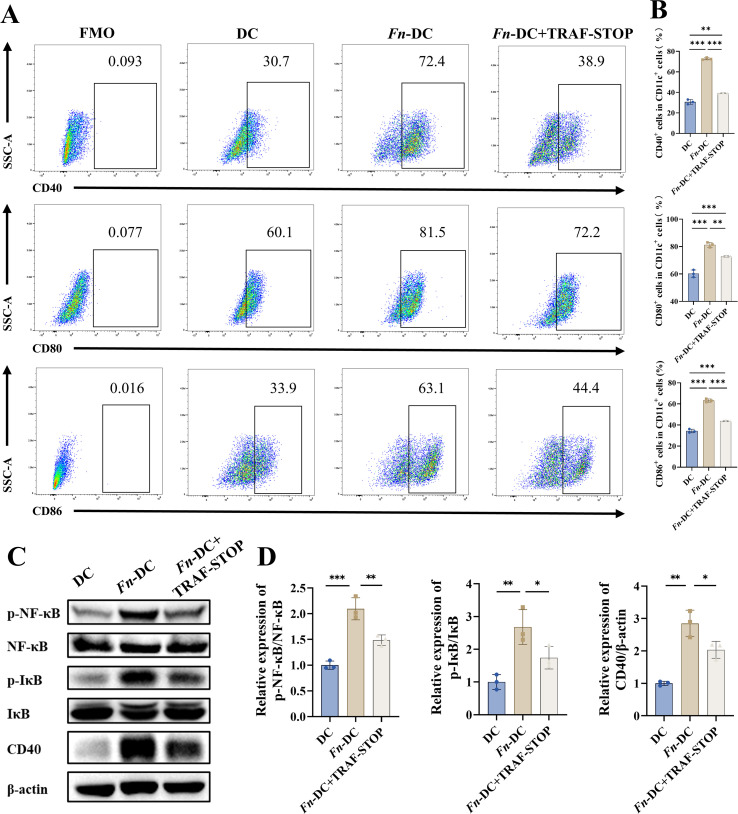
CD40 inhibition suppresses *Fn*-induced dysregulated activation of DCs. **(A)** Representative flow cytometry plots showing CD40^+^, CD80^+^, and CD86^+^ expression on CD11c^+^ DCs. **(B)** Quantification of the percentage of CD40^+^, CD80^+^, and CD86^+^ DCs from **(A)**, with statistical comparisons between groups (n=3). **(C)** Representative Western blot showing protein expression levels of CD40 and the phosphorylation of downstream pathway. **(D)** Quantification of protein expression levels shown in **(C)**, with statistical comparisons between groups (n=3). *P < 0.05, **P < 0.01, ***P < 0.001; ns, not significant.

To investigate the immunomodulatory effect of CD40 signaling in *Fn*-exacerbated inflammation, BMDCs were co-cultured with *Fn* and treated with a selective CD40 inhibitor (TRAF-STOP). Flow cytometry revealed that TRAF-STOP markedly reduced *Fn*-induced CD40^+^ DCs and concomitantly suppressed CD80/CD86 expression (**Figures 6A, B**). Western blot showed that TRAF-STOP attenuated *Fn*-induced CD40 protein expression and decreased phosphorylation of downstream signaling molecules (**Figures 6C, D**). These findings confirm that *Fn* activates DCs primarily through CD40 signaling, as blocking this axis effectively reversed the pro-inflammatory phenotype.

**Figure 7:**
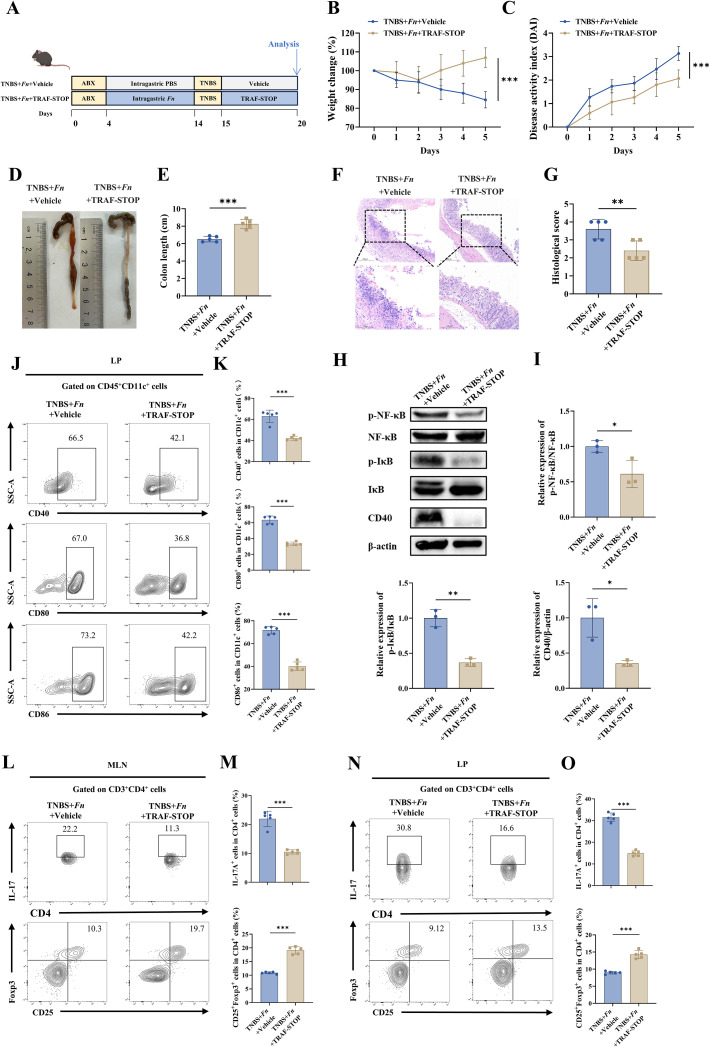
CD40 inhibition with TRAF-STOP attenuates DC activation, restores Th17/Treg balance, and ameliorates colitis in mice. **(A)** The schematic demonstrates the experiment procedures (n=5 mice per group). **(B)** Mice were weighed daily. **(C)** Disease activity index (DAI) for experimental colitis mice. The general appearance **(D)** and lengths of colons **(E)** in each group. **(F)** Representative images of H&E staining of colon sections, scale bar = 200 μm (above row) and scale bar = 100 µm (below row), and histological scores **(G)** were analyzed. **(H)** Representative Western blot showing the expression levels of CD40 and the phosphorylation of downstream pathway in colonic tissues. **(I)** Densitometric analysis of protein expression from **(H)**, with statistical comparisons between groups. **(J)** Representative flow cytometry plots showing the expression of CD40, CD80, and CD86 on CD11c^+^ DCs within colonic LPMCs. **(K)** Quantification of CD40^+^, CD80^+^, and CD86^+^ cells among CD11c^+^ DCs from **(J)**, presented as bar graphs. **(L)** Representative flow cytometry plots showing the expression of IL-17A^+^ or CD25^+^Foxp3^+^ on CD4^+^ T cells within MLNs. **(M)** Quantification of IL-17A^+^ or CD25^+^Foxp3^+^ cells among CD4^+^ T cells from **(L)**, presented as bar graphs. **(N)** Representative flow cytometry plots showing the expression of IL-17A^+^ or CD25^+^Foxp3^+^ on CD4^+^ T cells within LP. **(O)** Quantification of IL-17A^+^ or CD25^+^Foxp3^+^ cells among CD4^+^ T cells from **(N)**, presented as bar graphs. *P < 0.05, **P < 0.01, ***P < 0.001; ns, not significant.

To further examine the contribution of CD40 signaling to *Fn*-mediated colitis *in vivo*, we employed the TNBS-induced murine model with *Fn* gavage and TRAF-STOP intervention. During TNBS challenge, mice received daily intraperitoneal injections of vehicle or TRAF-STOP (**Figure 7A**). Notably, pharmacological inhibition of CD40 signaling significantly attenuated *Fn*-aggravated colitis, as evidenced by less body weight loss (**Figure 7B**), reduced DAI scores (**Figure 7C**), preserved colon length (**Figures 7D, E**), and improved histological features (**Figures 7F, G**). These results, together with *in vitro* data, provide compelling evidence that *Fn* exacerbates colonic inflammation under TNBS-induced conditions predominantly via CD40 signaling.

Building on these findings, we investigated how CD40 inhibition modulates DC activation and downstream signaling within colonic LP. TRAF-STOP treatment markedly reduced CD40^+^ DCs and simultaneously suppressed CD80/CD86 expression (**Figures 7J, K**). Western blot demonstrated decreased CD40 expression and reduced phosphorylation of downstream signaling molecules following TRAF-STOP (**Figures 7H, I**), indicating effective suppression of CD40-mediated DC activation. We next assessed Th17/Treg differentiation in MLNs and colonic LP. As expected, TRAF-STOP treatment significantly reduced Th17 cells while increasing Treg cells, thereby restoring the Th17/Treg balance upon CD40 pathway blockade (**Figures 7L-O**).

**Figure 8:**
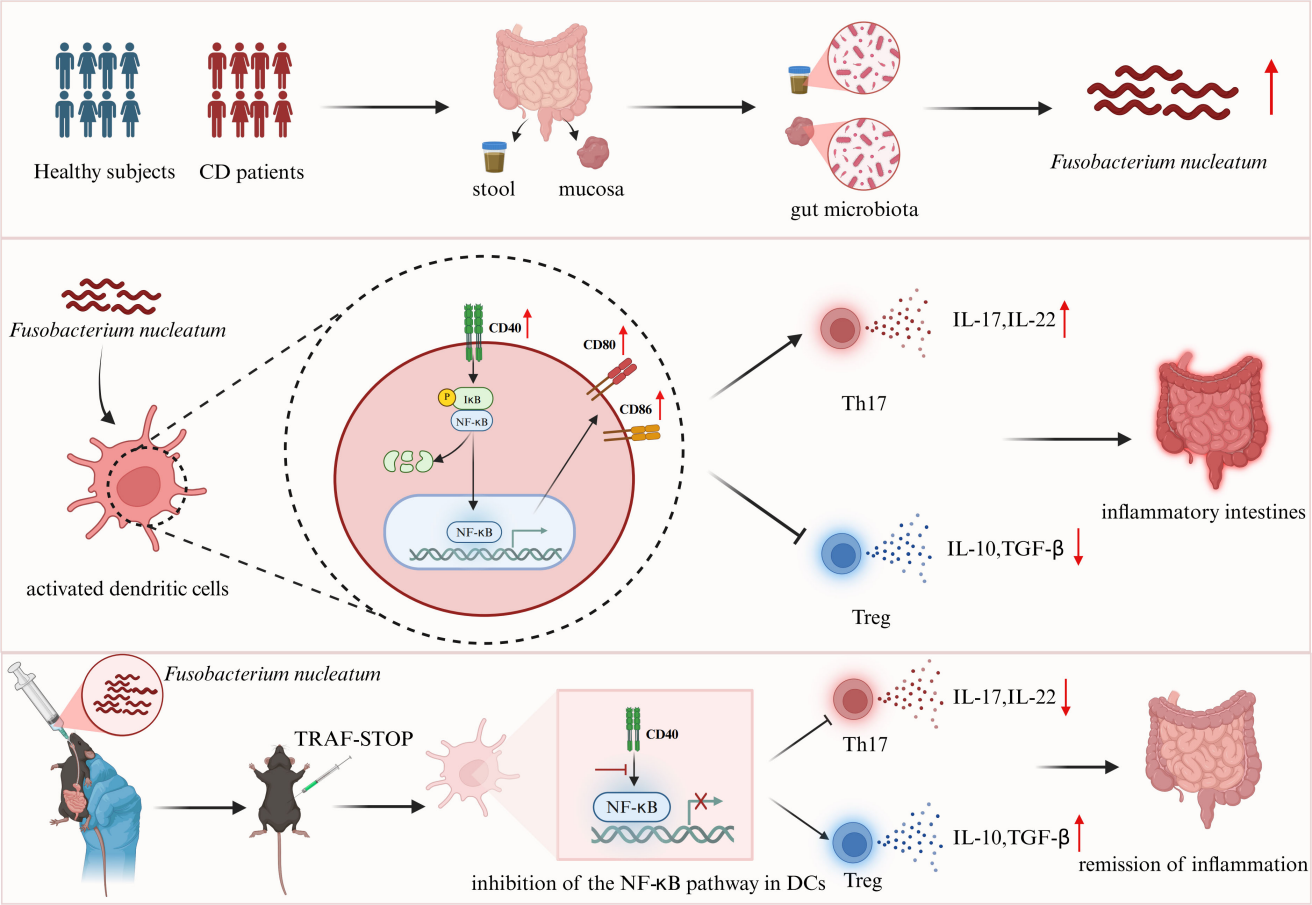
Schematic model of the *Fn*–CD40–DC axis exacerbating intestinal inflammation. *Fn* enrichment in CD activates mucosal DCs via CD40–NF-κB signaling under inflammatory conditions, enhancing co-stimulatory molecule expression and skewing Th17/Treg differentiation toward a pro-inflammatory state. CD40 inhibition reverses DC hyperactivation and restores immune balance, leading to amelioration of intestinal inflammation.

Collectively, our data demonstrate that *Fn* exacerbates intestinal inflammation under inflammatory conditions in CD via CD40-mediated DC activation and subsequent Th17/Treg imbalance. CD40 signaling inhibition with TRAF-STOP effectively restores DC homeostasis and Th17/Treg balance, highlighting CD40 as a targetable mediator linking microbial dysbiosis to mucosal immune dysregulation (**Figure 8**).

## Discussion

Crohn’s disease is a complex, chronic inflammatory disorder of the gastrointestinal tract, characterized by progressive intestinal damage and immune dysregulation (4, 32). Microbial dysbiosis is now recognized as a key factor in the pathogenesis of CD, with emerging evidence supporting a direct link between gut microbial imbalance and inflammation (33). In this study, we identified *Fn*, a bacterium previously implicated in colorectal cancer (5, 6, 34–36), as a critical contributor to the exacerbation of gut inflammation in CD. This finding provides additional evidence supporting the pathogenic role of gut microbial dysbiosis in CD and underscore the relevance of specific microbial species in shaping disease progression. These observations are consistent with the multi-omics study by Li et al., which analyzed the fecal microbiome of 278 CD patients and 28 healthy controls and demonstrated a significant increase in *Fusobacterium* abundance in CD (3). It should be noted that, to minimize demographic bias, baseline characteristics were comparable between CD patients and healthy controls. Within this cohort, the potential impact of demographic variables on microbial composition could not be evaluated. We acknowledge that demographic factors such as age and sex likely shape the gut microbiota, and this warrants further investigation in future studies.

In the context of infection, APCs, such as DCs, phagocytose bacteria, migrate to lymph nodes, and present antigens to naïve T cells (37). Growing evidence supports that mature DCs, characterized by elevated CD80/CD86 expression, drive intestinal inflammation by secreting pro-inflammatory cytokines and disrupting the Th17/Treg balance (12, 13, 38). Based on these observations, we investigated the impact of *Fn* in a TNBS-induced murine colitis model and found that *Fn* markedly increased CD80^+^CD86^+^ DCs in the colonic LP and induced a Th17/Treg imbalance in MLNs. To further validate the direct effect of *Fn* on DCs, we co-cultured BMDCs with *Fn*, which resulted in elevated CD80/CD86 expression, supporting the hypothesis that *Fn* directly enhances DC maturation and contributes to intestinal inflammation under inflammatory conditions. Therefore, we adoptively transferred *Fn*-DCs into mice and observed exacerbated inflammation, which was accompanied by a disrupted Th17/Treg balance, compared to mice receiving untreated BMDCs or PBS. Despite these robust findings, it is important to acknowledge certain limitations associated with the use of heat-inactivated *Fn* in our *in vivo* experiments. To mitigate the risk of bacteremia and systemic infection, particularly in adoptive transfer experiments, we used heat-inactivated *Fn*. Live and heat-inactivated bacteria, however, differ in immunogenic potential: live organisms can colonize and continuously release metabolites, eliciting stronger and more sustained immune activation (39), whereas heat inactivation abrogates viability and may alter surface structures (e.g., membrane proteins and polysaccharides), thereby modifying host-microbe interactions (40, 41). Nonetheless, certain heat-stable pathogen-associated molecular patterns, most notably lipopolysaccharide (LPS), retain bioactivity after heat inactivation and can drive a pro-inflammatory DC phenotype (41). Thus, while heat-inactivated *Fn* cannot fully reproduce the dynamic responses elicited by live bacteria, it can partially recapitulate key inflammatory signals. Consistent with this, our data show that even without viability, *Fn* modulates DC activation and disrupts the Th17/Treg balance, thereby aggravating intestinal inflammation.

We further investigated the role of *Fn* in modulating DC activation via CD40 signaling, which was identified as a key mediator of *Fn*-DC interactions by RNA-seq. Our study provides novel insights into the direct activation of CD40 signaling by *Fn* in DCs, positioning CD40 as a pivotal link between microbial dysbiosis and mucosal immune modulation. While CD40 overexpression has been recognized as a hallmark of pro-inflammatory signaling in CD (19, 20), its association with gut dysbiosis, particularly *Fn* enrichment, remains unexplored. Our findings show that *Fn* significantly upregulated CD40 expression in intestinal DCs and activated the NF-κB pathway, concomitant with exacerbated gut inflammation in a TNBS-induced murine colitis model. Notably, pharmacological inhibition of CD40 using TRAF-STOP not only effectively suppressed *Fn*-induced NF-κB activation and ameliorated colitis severity, but also restored immune balance by reducing Th17 cells and increasing Treg cells. We acknowledge that TNBS-induced colitis represents an acute inflammation model and thus cannot fully reflect the chronic and relapsing course of CD. Nonetheless, TNBS is widely employed to study dendritic cell activation and T cell imbalance *in vivo* (42). Mechanistically, it induces IL-12-mediated, Th1-driven transmural colitis, closely resembling human IBD at both histological and immunological levels. On this basis, TNBS provides a reliable system for mechanistic investigation in our study (43). Future work employing chronic colitis models, such as DSS-induced colitis or IL-10-deficient mice, will be essential to validate and extend these conclusions. In addition, studies incorporating high-dimensional immune profiling of human intestinal samples, such as single-cell transcriptomics, will help further refine the translational relevance of the CD40–DC axis.

Beyond the preclinical relevance, our findings carry important translational implications. Recent clinical studies have demonstrated the feasibility and safety of CD40 blockade in humans. A first-in-human PhaseI trial of the anti-CD40 monoclonal antibody KPL-404 revealed favorable tolerability, complete receptor occupancy, and suppression of T-cell–dependent antibody responses in healthy volunteers, suggesting that targeted CD40 inhibition can modulate immune responses without inducing broad immunosuppression (44). Similarly, a clinical study using ch5D12, another antagonistic anti-CD40 antibody, in patients with moderate-to-severe Crohn’s disease showed promising safety and pharmacodynamic profiles (45). These studies support the clinical applicability of CD40-targeted strategies and underscore the therapeutic relevance of our findings. Importantly, CD40 blockade could serve as a complementary approach to existing therapies. In particular, combining CD40 inhibition with anti-TNF or anti-IL-23 agents may achieve synergistic effects by simultaneously targeting innate and adaptive immune pathways, thereby offering novel therapeutic avenues for CD patients.

Taken together, our findings uncover a novel mechanism whereby *Fn* exacerbates gut inflammation under inflammatory conditions relevant to CD through CD40-mediated DC activation and downstream Th17/Treg imbalance. Importantly, pharmacological inhibition of CD40 restored immune homeostasis and ameliorated colitis severity, underscoring the immunoregulatory potential of targeting this pathway. This study highlights a previously unrecognized microbial–immune crosstalk in CD, wherein *Fn*-driven DC activation orchestrates pathogenic adaptive immune polarization. Targeting this axis offers a promising microbiota-directed immunomodulatory strategy to improve therapeutic outcomes in CD.

## Data Availability

The datasets presented in this study can be found in online repositories. The names of the repository/repositories and accession number(s) can be found below: https://ngdc.cncb.ac.cn/search/specific?db=hra&q=PRJCA039780,PRJCA039780https://ngdc.cncb.ac.cn/search/specific?db=hra&q=PRJCA039546,PRJCA039546.
