# Supplementary material for: Fusobacterium nucleatum drives CD40-mediated dendritic cell activation and Th17/Treg imbalance to exacerbate intestinal inflammation in Crohn’s disease
Source: Front Immunol. 2026 Jan 6;16:1712971. doi: 10.3389/fimmu.2025.1712971 (PMC12815848; doi:10.3389/fimmu.2025.1712971)
Supplement: Supplementary file 1 [file Table1.docx]

**Table S1: Clinical information on participants**

| **Indicators** | **CD patients**  **(n=24)** | **Healthy subjects**  **(n=15)** | ***P.Value*** |
| --- | --- | --- | --- |
| Gender |  |  |  |
| Male, n (%)  Female, n (%) | 15 (62.50%)  9 (37.50%) | 9 (60.00%)  6 (40.00%) | 0.8338 |
| Age, Median (Q1, Q3) | 30.00 (26.00-41.00) | 33.00 (26.75-40.75) | 0.559 |
| BMI, Median (Q1, Q3) | 19.47 (18.47-21.47) | 20.16 (19.48-21.95) | 0.062 |
| Smoking history or current smoking, n (%) | 4 (16.67%) | 3 (20%) | 0.425 |
| Age at diagnosis (Montreal classification), n (%)  ≤16 (A1)  17–40 (A2)  >40 (A3) | 0 (0%)  18 (75.00%)  6 (25.00%) | N/A | N/A |
| Location (Montreal classification), n (%)  ileal (L1)  colonic (L2)  ileocolonic (L3)  upper gastrointestinal disease (L4) | 7 (29.17%)  1 (4.17%)  15 (62.5%)  1 (4.17%) | N/A | N/A |
| Behavior (Montreal classification), n (%)  non-stricturing, non-penetrating (B1)  structuring (B2)  penetrating (B3)  perianal disease modifier (p) | 12 (50.00%)  10 (41.66%)  2 (8.33%)  11 (45.83%) | N/A | N/A |
| Simple Endoscopic Score for Crohn's Disease (SES-CD), mean ± SD | 18.50±7.93 | N/A | N/A |
| Crohn′s disease activity index (CDAI), mean ± SD | 285.47±91.31 | N/A | N/A |
| Fecal and serum inflammatory markers |  | N/A | N/A |
| fecal calprotectin (FC), μg/g, mean ± SD  C-reactive protein (CRP), mg/L, mean ± SD  erythrocyte sedimentation rate (ESR), mm/h, mean ± SD  white blood cell count (WBC), 10^9/L, mean ± SD  platelets (PLT), 10^9/L, mean ± SD  packed cell volume (PCV), %, mean ± SD | 938.81±394.61  21.61±17.73  41.75±25.85  6.12±1.33  299.54±96.35  37.86±5.49 |  |  |
| serum albumin (ALB), g/L, mean ± SD | 36.04±6.32 |  |  |

**Table S2: Clinical information of GEO data**

| **Accession** | **Source** | **Location** | **Indicators** | **CD patients** | **Control** |
| --- | --- | --- | --- | --- | --- |
| GSE75214 | Mucosa | Colon | Gender | 8 | 11 |
|  |  |  | Male, n (%)  Female, n (%)  Age, Median (Q1, Q3) | N/A -  N/A  N/A | N/A  N/A  N/A |
|  |  |  |  |  |  |
| GSE261086 | Mucosa | Colon | Gender  Male, n (%)  Female, n (%)  Age, Median (Q1, Q3) | 6  N/A  N/A  N/A | 6  N/A  N/A  N/A |
| GSE52746 | Mucosa | Colon |  | 10 | 17 |
|  |  |  | Gender |  |  |
|  |  |  | Male, n (%)  Female, n (%) | 7 (70%)  3 (30%) | 10 (58.83%)  7 (41.18%) |
|  |  |  | Age, Median (Q1, Q3) | 34.0 (33.0-41.5) | 50.0 (39.0-54.0) |

**Table S3: The sequences of gene-specific primer sets used for qPCR**

| **Oligonucleotides** | **SOURCE** | **IDENTIFIER** |
| --- | --- | --- |
| CD40-F: ACTGAAACGGAATGCCTTCCT | Tsingke | N/A |
| CD40-R: CCTCACTCGTACAGTGCCA |  |  |
| GAPDH-F: CACCATCTTCCAGGAGCGAG | Tsingke | N/A |
| GAPDH-R: GATGGCATGGACTGTGGTCA |  |  |
| mCd40-F: TGTCATCTGTGAAAAGGTGGTC | Tsingke | N/A |
| mCd40-R: ACTGGAGCAGCGGTGTTATG |  |  |
| mGapdh-F: AGGTCGGTGTGAACGGATTTG | Tsingke | N/A |
| mGapdh-R: TGTAGACCATGTAGTTGAGGTCA |  |  |
| F. nucleatum-F: CAACCATTACTTTAACTCTACCATGTTCA | Tsingke | N/A |
| F. nucleatum-R: GTTGACTTTACAGAAGGAGATTATGTAAAAATC |  |  |
| 16S-F: GTGCCAGCMGCCGCGGTAA | Tsingke | N/A |
| 16S-R: GACTACCAGGGTATCTAATCC |  |  |

**Figure S1: Gating strategies**

**
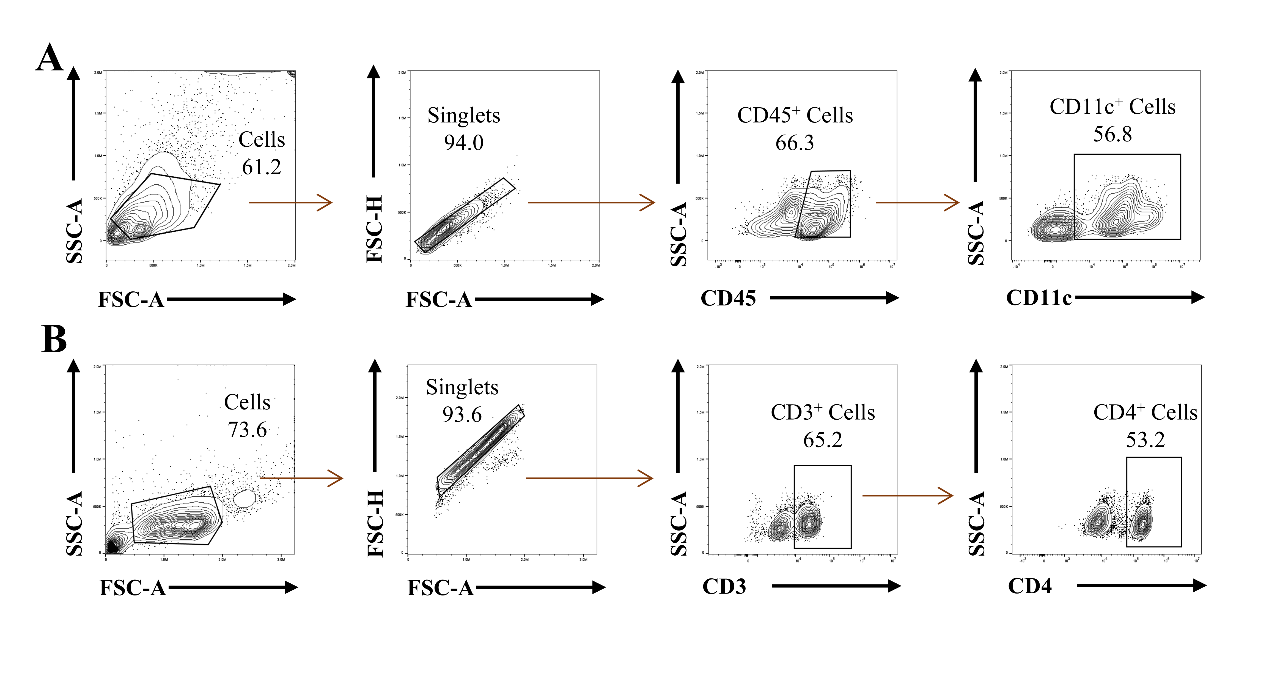
**

Related to Figures 2, 3, 4, 5, 7. (A) Gating strategy pertaining to Figures 2 and 5. (B) Gating strategy pertaining to Figures 3, 4, and 7.

**Figure S2: *Fusobacterium nucleatum* stimulates DCs *in vitro* to transform into an inflammatory phenotype
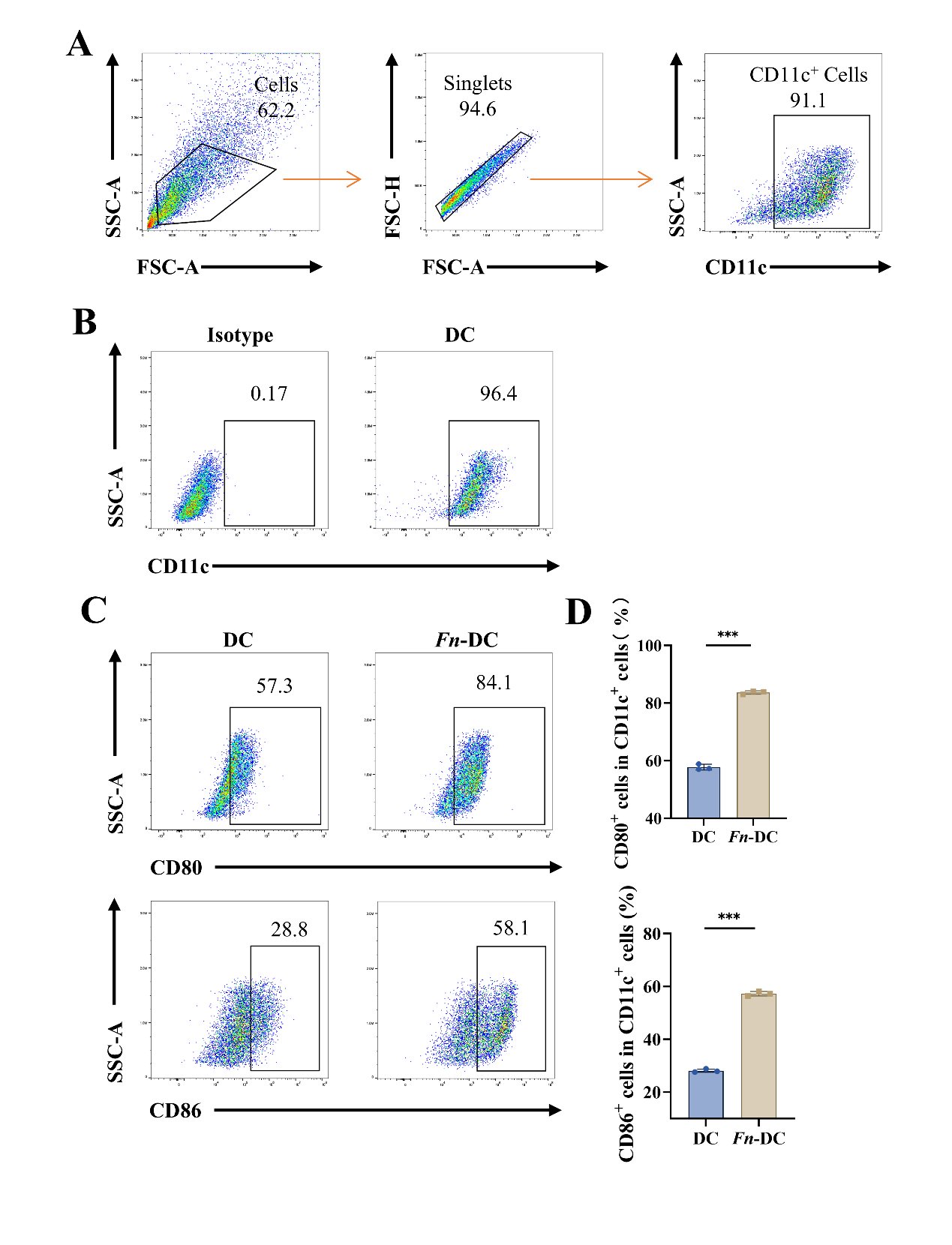
** (A) Gating strategy pertaining to Figures 6 and S2. (B) Detection of the purity of dendritic cells. (C) Representative flow cytometry plots showing CD80⁺ and CD86⁺ expression on CD11c⁺ DCs. (D) Quantification of CD80⁺ and CD86⁺ cells among CD11c⁺ DCs, shown as bar graphs. **P* < 0.05, ***P* < 0.01, ****P* < 0.001; ns: not significant.
